# Left ventricular assessment with artificial intelligence increases the diagnostic accuracy of stress echocardiography

**DOI:** 10.1093/ehjopen/oeac059

**Published:** 2022-09-21

**Authors:** Jamie M O’Driscoll, William Hawkes, Arian Beqiri, Angela Mumith, Andrew Parker, Ross Upton, Annabelle McCourt, William Woodward, Cameron Dockerill, Nikant Sabharwal, Attila Kardos, Daniel X Augustine, Katrin Balkhausen, Badrinathan Chandrasekaran, Soroosh Firoozan, Anna Marciniak, Stephen Heitner, Mrinal Yadava, Sanjiv Kaul, Rizwan Sarwar, Rajan Sharma, Gary Woodward, Paul Leeson

**Affiliations:** Ultromics Ltd, 4630 Kingsgate, Cascade Way, Oxford Business Park South, Oxford OX4 2SU, UK; School of Psychology and Life Sciences, Canterbury Christ Church University, North Holmes Road, Kent CT1 1QT, UK; Department of Cardiology, St George’s University Hospitals NHS Foundation Trust, Blackshaw Road, Tooting, London SW17 0QT, UK; Ultromics Ltd, 4630 Kingsgate, Cascade Way, Oxford Business Park South, Oxford OX4 2SU, UK; Ultromics Ltd, 4630 Kingsgate, Cascade Way, Oxford Business Park South, Oxford OX4 2SU, UK; Ultromics Ltd, 4630 Kingsgate, Cascade Way, Oxford Business Park South, Oxford OX4 2SU, UK; Ultromics Ltd, 4630 Kingsgate, Cascade Way, Oxford Business Park South, Oxford OX4 2SU, UK; Ultromics Ltd, 4630 Kingsgate, Cascade Way, Oxford Business Park South, Oxford OX4 2SU, UK; Cardiovascular Clinical Research Facility, Radcliffe Department of Medicine, Division of Cardiovascular Medicine, University of Oxford, John Radcliffe Hospital, Oxford OX3 9DU, UK; Cardiovascular Clinical Research Facility, Radcliffe Department of Medicine, Division of Cardiovascular Medicine, University of Oxford, John Radcliffe Hospital, Oxford OX3 9DU, UK; Cardiovascular Clinical Research Facility, Radcliffe Department of Medicine, Division of Cardiovascular Medicine, University of Oxford, John Radcliffe Hospital, Oxford OX3 9DU, UK; Cardiovascular Clinical Research Facility, Radcliffe Department of Medicine, Division of Cardiovascular Medicine, University of Oxford, John Radcliffe Hospital, Oxford OX3 9DU, UK; Oxford Heart Centre, Oxford University Hospitals NHS Foundation Trust, Oxford OX3 9DU, UK; Department of Cardiology, Milton Keynes University Hospital NHS Foundation Trust, Milton Keynes MK6 5LD, UK; Department of Cardiology, Royal United Hospitals NHS Foundation Trust, Bath BA1 3NG, UK; Department for Health, University of Bath, Bath BA2 7JU, UK; Department of Cardiology, Royal Berkshire NHS Foundation Trust, Reading RG1 5AN, UK; Department of Cardiology, Great Western Hospitals NHS Foundation Trust, Swindon SN3 6BB, UK; Department of Cardiology, Buckinghamshire Healthcare NHS Trust, High Wycombe HP7 0JD, UK; Department of Cardiology, St George’s University Hospitals NHS Foundation Trust, Blackshaw Road, Tooting, London SW17 0QT, UK; Knight Cardiovascular Institute, Oregon Health & Science University, Portland, OR 97239, USA; Knight Cardiovascular Institute, Oregon Health & Science University, Portland, OR 97239, USA; Knight Cardiovascular Institute, Oregon Health & Science University, Portland, OR 97239, USA; Ultromics Ltd, 4630 Kingsgate, Cascade Way, Oxford Business Park South, Oxford OX4 2SU, UK; Cardiovascular Clinical Research Facility, Radcliffe Department of Medicine, Division of Cardiovascular Medicine, University of Oxford, John Radcliffe Hospital, Oxford OX3 9DU, UK; Oxford Heart Centre, Oxford University Hospitals NHS Foundation Trust, Oxford OX3 9DU, UK; Experimental Therapeutics, Radcliffe Department of Medicine, University of Oxford, John Radcliffe Hospital, Oxford OX3 9DU, UK; Department of Cardiology, St George’s University Hospitals NHS Foundation Trust, Blackshaw Road, Tooting, London SW17 0QT, UK; Ultromics Ltd, 4630 Kingsgate, Cascade Way, Oxford Business Park South, Oxford OX4 2SU, UK; Cardiovascular Clinical Research Facility, Radcliffe Department of Medicine, Division of Cardiovascular Medicine, University of Oxford, John Radcliffe Hospital, Oxford OX3 9DU, UK

**Keywords:** Artificial intelligence, Stress echocardiography, Contrast, Coronary artery disease, Ejection fraction, Global longitudinal strain

## Abstract

**Aims:**

To evaluate whether left ventricular ejection fraction (LVEF) and global longitudinal strain (GLS), automatically calculated by artificial intelligence (AI), increases the diagnostic performance of stress echocardiography (SE) for coronary artery disease (CAD) detection.

**Methods and results:**

SEs from 512 participants who underwent a clinically indicated SE (with or without contrast) for the evaluation of CAD from seven hospitals in the UK and US were studied. Visual wall motion scoring (WMS) was performed to identify inducible ischaemia. In addition, SE images at rest and stress underwent AI contouring for automated calculation of AI-LVEF and AI-GLS (apical two and four chamber images only) with Ultromics EchoGo Core 1.0. Receiver operator characteristic curves and multivariable risk models were used to assess accuracy for identification of participants subsequently found to have CAD on angiography. Participants with significant CAD were more likely to have abnormal WMS, AI-LVEF, and AI-GLS values at rest and stress (all *P* < 0.001). The areas under the receiver operating characteristics for WMS index, AI-LVEF, and AI-GLS at peak stress were 0.92, 0.86, and 0.82, respectively, with cut-offs of 1.12, 64%, and −17.2%, respectively. Multivariable analysis demonstrated that addition of peak AI-LVEF or peak AI-GLS to WMS significantly improved model discrimination of CAD [C-statistic (bootstrapping 2.5th, 97.5th percentile)] from 0.78 (0.69–0.87) to 0.83 (0.74–0.91) or 0.84 (0.75–0.92), respectively.

**Conclusion:**

AI calculation of LVEF and GLS by contouring of contrast-enhanced and unenhanced SEs at rest and stress is feasible and independently improves the identification of obstructive CAD beyond conventional WMSI.

## Graphical Abstract

**Graphical Abstract:**
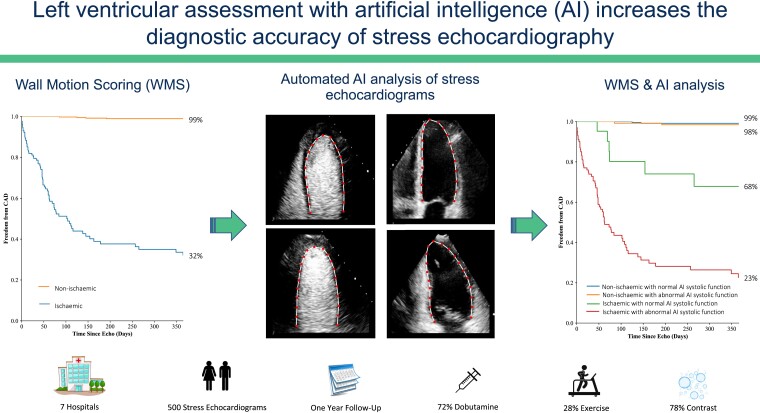

## Introduction

Stress echocardiography (SE) is one of the most widely performed, accessible, and cost-effective procedures for both the diagnosis and risk stratification of patients with known or suspected coronary artery disease (CAD).^1^ Although wall motion scoring (WMS) of SEs by expert readers has a high accuracy for identifying CAD, the inherent reliance on this subjective assessment can lead to significant variability in accuracy between centres and operators.^2^

Recent meta-analysis has demonstrated that use of quantitative measures of left ventricular systolic function, including left ventricular ejection fraction (LVEF) and global longitudinal strain (GLS) may provide similar diagnostic accuracy for CAD on stress echocardiography (SE) as WMS.^3,4^ However, the benefit of quantitative measurements has not yet been translated in routine clinical practice. This is, in part, because GLS measurement by speckle tracking is less accurate during the tachycardia induced by SE.^5^

We have recently reported on an artificial intelligence (AI) image processing pipeline that has been trained to analyse echocardiograms acquired during SE.^6^ The pipeline contours the left ventricular (LV) in all frames and can calculate LVEF and GLS (apical two and four chamber images only) automatically, with zero measurement variability, unlike manual contouring. As the pipeline has been trained on SE it is also able to calculate LVEF and GLS in the presence of contrast enhancement and over a wide range of heart rates. The reduced variability in measurement of LVEF and GLS introduced by the automated approach has so far been shown to improve precision for identification of left ventricular changes in COVID-19 infection^7^ and cancer patients.^8^

We hypothesise that automated AI-calculated LVEF and GLS extracted from a large number of real-world SEs at rest and stress adds value to WMS for identification of patients with CAD.

## Method

### Study design

The study data set consisted of 512 consecutive participants fulfilling the exclusion and inclusion criteria of the study who underwent a clinically indicated SE for investigation of chest pain from six UK hospitals (see Supplementary material online, *Figure S1* for study flow diagram). Of these, 12 sets of images were not interpretable resulting in a final sample size of 500 (*n* = 362; Buckinghamshire Healthcare NHS Trust; Great Western Hospitals NHS Foundation Trust; Milton Keynes University Hospital NHS Foundation Trust; John Radcliffe Hospital, Oxford; Royal United; Royal Berkshire NHS Foundation Trust; St George’s University Hospitals NHS Foundation Trust, London) from the multicentre prospective EVAREST study (Echocardiography Value and Accuracy at REst and Stress; ClinicalTrials.gov identifier:NCT03674255)^1,6^ and the retrospective RAINIER study (*n* = 138) from the Oregon Health & Science University Hospital (OHSU, Portland, Oregon, United States of America).^6^ This is an independent, retrospective sub-study using a previously reported data set.^6^ Exclusion criteria included sub-maximal haemodynamic stress in the absence of wall motion abnormalities (defined as not reaching the target heart rate or rate-pressure product depending on the mode of stress), previous coronary revascularization, previous myocardial infarction, asymptomatic patients awaiting non-cardiac surgery, participants referred only for the assessment of myocardial viability and severity of valvular heart disease. This investigation conformed to the Declaration of Helsinki principles; the Health Research Authority National Research Ethics Service Committee South Central–Berkshire gave ethical approval for the EVAREST study (IRAS ID:162119), and a waiver for consent for the RAINIER study was given by the OHSU Institutional Review Board.

### Stress echocardiography

All participants underwent a dobutamine or exercise SE, either with or without ultrasound contrast agents according to local protocols and the operators’ decisions. SEs were performed either using Phillips (iE33 and EPIQ 7C) or GE (Vivid E95) echocardiography machines, and included acquisition of the parasternal short-axis and the apical four-, three-, and two-chamber (A4C, A2C, and A3C, respectively) views. The target heart rate for dobutamine SEs was defined as 85% of the age-predicted maximal heart rate, which itself was calculated as 220-age; rate-pressure product was calculated by multiplying the heart rate with the systolic blood pressure at peak stress, with a target of 20 000 beats mmHg/min.

### Visual scoring of wall motion

Semi-quantitative visual scoring of wall motion at rest and peak stress was calculated from the routine clinical reports by J.M.O. who was blinded to the clinical outcome and the results of the automated AI quantification (see Supplementary material online, *Table S1*). WMS was performed on a 4-point scale (1, normal wall motion; 2, hypokinesis; 3, akinetic; 4, dyskinetic) and used to calculate the wall motion score index (WMSI) at rest and peak stress.^9^ An abnormal (ischaemic) response was defined as the worsening of wall motion under stress compared to resting function, and the ischaemic burden was categorized as low (1–2 ischaemic LV segments) or moderate to severe (≥3 ischaemic LV segments).^10^

### Automated artificial intelligence calculation of left ventricular ejection fraction and global longitudinal strain from EchoGo

**Figure 1:**
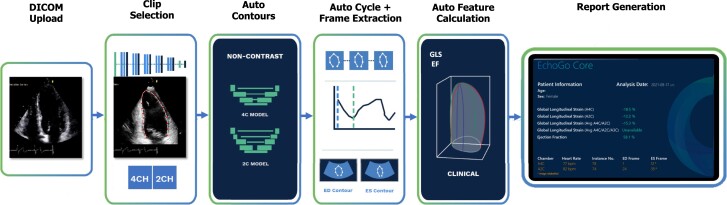
Artificial intelligence data flow. EchoGo Core is an automated, cloud-based software medical device for processing of echocardiographic images. Echocardiographic images are uploaded to the cloud-based environment in DICOM format whereby an automated pipeline identifies the apical two and four chamber images available for analysis. Trained and accredited cardiac physiologists (operators) review the identified images and ensure image selection is appropriate. Apical images are then passed to convolutional neural networks which delineate the left ventricular endocardium, predict the position of endocardial segments using the 18-segment model and then contour the endocardial border. Operators conduct a quality control check of all contours produced by the software by either accepting or rejecting them. Operators are unable to manually edit or adjust contours but they are able to select available alternative images for auto-contouring. Left ventricular global longitudinal strain is calculated as the average Lagrangian strain from contours of the apical two and four chamber images. Left ventricular volumes and ejection fraction are calculated using the apical two and four chamber contours using the Simpson’s biplane method.

All SE images were analysed with EchoGo Core 1.0 (Ultromics, Oxford) as previously described.^6^ In brief, the AI algorithm was used for automated contouring of the endocardial border of every frame from the A4C and A2C views, automated identification of the end-diastolic and the end-systolic frames based upon the size of the enclosed area, and automated selection of the cardiac cycle (*Figure 1*). The automated LV contours and selection of frames were verified and approved by at least one accredited echocardiographer who was blinded to all other clinical information. Entire SE studies were only approved for analysis if the entire LV endocardial border was adequately delineated by the AI from all views. LVEF was measured using the modified Simpson’s biplane method of discs and GLS was measured using the average longitudinal strain from the A4C and A2C views. The augmentation in WMSI, LVEF, and GLS during stress, ΔWMSI, ΔLVEF, and ΔGLS, respectively, were calculated by subtracting the values at peak from those at rest. Ischaemic dilatation ratios were calculated by dividing the LV volumes at peak stress by those at rest.

### Participant follow-up and outcomes

For all SE studies, clinical follow-up information for 12 months after the SE was obtained from blinded review of medical records. The primary end-point of interest was clinically significant CAD on coronary angiography performed electively or on an emergent basis. CAD was defined as ≥50% left main CAD and/or ≥70% stenosis in any of the proximal to mid left anterior descending, or proximal left circumflex or right coronary artery, as well as the mid-right coronary artery if the circulation was right dominant.^9^ The clinical management of patients who underwent coronary angiography was recorded, and those who were managed medically without further acute event or requirement for investigation were deemed to have been managed appropriately. Disease classifications were determined by Adjudication Committees, comprising at least one board certified cardiologist, with all members of the committees being blinded to the SE results.

### Statistical analysis

Unless otherwise specified, data are presented as mean ± standard deviation or *n* (%). Group comparisons were performed using the Student’s *t*-test for continuous data and categorical data were compared with *χ*^2^-test or Fisher’s exact test, as appropriate. The optimal cut-off values for LVEF and GLS was determined from the area under the receiver operator characteristic curve (ROC, AUROC) analysis based upon optimization of sensitivity and specificity by calculating Youden’s index. For brevity and simplicity, participants with AI quantification of LVEF and GLS, respectively less than or greater than the cut-off thresholds were classed as having abnormal AI-calculated systolic function, and normal AI-calculated systolic function otherwise, rather than being classified as ‘not abnormal.’

Kaplan–Meier event curves were constructed and compared using the log-rank test to allow evaluation of both diagnostic outcome and time to diagnosis. Patients who underwent angiography during follow-up and were confirmed to have non-significant CAD were right-censored. In addition, patients without an event >12-months were right-censored at the end of the follow-up period. The data were stratified according to AI-calculated peak LVEF, AI-calculated GLS, the presence of inducible ischaemia, or a combination thereof. Logistic regression was conducted using Python (Version: 3.9.7) StatsModels and SKlearn packages. Univariable associations with CAD diagnosis were assessed without adjustment for potential confounders and included, age, sex, cardiovascular risk factors (hypertension, diabetes mellitus, hypercholesterolaemia), inducible ischaemia, and AI-calculated LVEF and GLS. These simple models were generated to evaluate the odds ratios of individual variables and identify independent predictors of prognostically significant CAD following SE. Multivariable logistic regression was used to further investigate whether univariable associations hold true after accounting for confounding variables. Multicollinearity was assessed via calculation of the variance inflation factor. Variable selection for multivariable models was conducted using forward stepwise regression using a cut-off of 0.05 for *P*-values. Furthermore, multivariable logistic regression was implemented to determine the added value of metrics produced by our AI pipeline, when considered alongside risk factors and standard observations made by clinicians during the SE examination, and the *C*-statistic was calculated as a measure of the incremental value of AI-calculated LVEF and GLS. The *C*-statistic was estimated with bootstrapping, whereby 70% of the data was resampled, the logistic regression model was fit and then tested on the remaining 30% of the data over 1000 iterations. *C*-statistics are reported as the mean of the 1000 iterations with the distribution from 2.5th to 97.5th percentiles. A *P*-value < 0.05 was reported as statistically significant.

## Results

### Study population, procedures, and outcomes

SEs from a total of 500 participants were included in the analysis, with the majority having been performed with dobutamine (72%) rather than exercise (28%) stress, and the majority enhanced with contrast (78%). Most participants did not undergo coronary angiography (76%) during the follow-up period (see Supplementary material online, *Table S2*); of the 118 (24%) that did undergo coronary angiography, 25% had no significant CAD, 29% had single vessel disease, 25% had two vessel disease and 8% had three vessel disease. Of the 74 participants with significant CAD, 57% underwent percutaneous coronary intervention at the same setting, 16% were referred for surgical revascularization, 11% of angiograms were reviewed by the Heart Team, with the remaining 16% being managed medically. Participants with significant CAD were more likely to be older (*P* < 0.002), male (*P* < 0.001), and have the risk factors hypertension (*P* < 0.048), diabetes mellitus (*P* < 0.001), and hypercholesterolaemia (*P* < 0.001, *Table 1*).

**Table 1:** Clinical features of the study population and echocardiographic according to the presence of significant coronary artery disease

			No CAD (*n* = 426)	Significant CAD (*n* = 74)	*P*-value
**Demographics**	Age (years)		63 ± 12	67 ± 10	**0.002**
	Male sex, *n* (%)		177 (42%)	52 (70%)	**<0.001**
**Risk factors**	Hypertension (%)		189 (44%)	42 (57%)	**0.048**
	Diabetes mellitus (%)		67 (16%)	28 (38%)	**<0.001**
	Hypercholesterolaemia (%)		99 (23%)	41 (55%)	**<0.001**
**Chest pain and echocardiographic changes during SE**	Chest pain, *n* (%)		17 (4%)	16 (22%)	**<0.001**
	Wall motion abnormality, *n* (%)	fixed	28 (7%)	23 (31%)	**<0**.**001**
		inducible	60 (14%)	70 (95%)	**<0**.**001**
	Ischaemic LV segments	0	366 (86%)	5 (7%)	**<0**.**001**
		1–2	22 (5%)	9 (12%)	
		≥3	38 (9%)	60 (81%)	
	WMSI	rest	1.02 ± 0.11	1.11 ± 0.24	**<0**.**001**
		peak	1.04 ± 0.15	1.45 ± 0.30	**<0**.**001**
	Normal wall motion at rest and/or peak, *n* (%)	rest	401 (94%)	53 (72%)	**<0**.**001**
		both rest and peak	354 (83%)	4 (5%)	**<0**.**001**
	EDV (ml)	rest	110 ± 30	128 ± 38	**<0**.**001**
		peak	88 ± 29	116 ± 40	**<0**.**001**
	ESV (ml)	rest	42 ± 18	59 ± 33	**<0**.**001**
		peak	27 ± 15	54 ± 30	**<0**.**001**
	LVEF (ml)	rest	62 ± 9	57 ± 13	**<0**.**001**
		peak	70 ± 9	55 ± 12	**<0**.**001**
	GLS (%)	rest	−16.8 ± 4.5	−15.2 ± 5.0	**0**.**005**
		peak	−19.6 ± 5.1	−13.4 ± 4.3	**<0**.**001**
	Ischaemic dilatation ratio	end-diastolic	0.82 ± 0.21	0.92 ± 0.21	**<0**.**001**
		end-systolic	0.66 ± 0.27	0.98 ± 0.31	**<0**.**001**

Bold values represent statistically significant differences between groups. WMSI, wall motion score index; EDV, end-diastolic volume; ESV, end-systolic volume; LVEF, LV ejection fraction; GLS, global longitudinal strain.

### Changes in haemodynamics, wall motion score, and symptoms during stress

**Figure 2:**
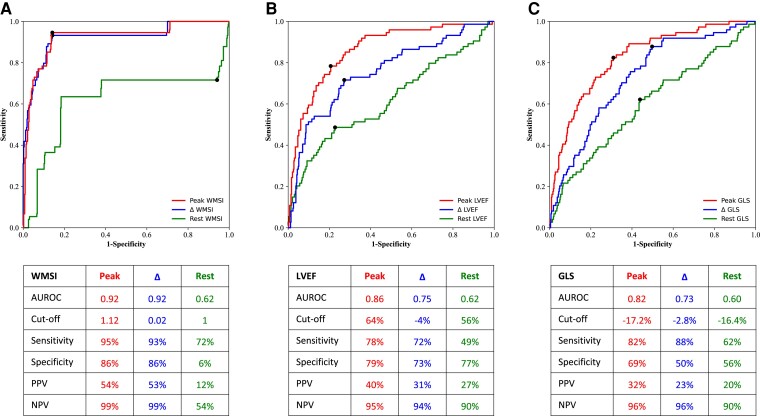
Receiver operator characteristic curves for the identification of significant coronary artery disease for peak, Δ and rest for (*A*) wall motion score index, (*B*) left ventricular ejection fraction, and (*C*) global longitudinal strain. Inset tables show the area under the receiver operator characteristic curve, optimal cut-off, sensitivity, positive predictive value, and negative predictive value for each receiver operator characteristic curve.

Participants with and without significant CAD had similar baseline haemodynamics and appropriate increases in their haemodynamic responses with both dobutamine and exercise stress (see Supplementary material online, *Table S3*). Participants with significant CAD were much more likely to develop chest pain with stress and have wall motion abnormalities at rest and with stress (all *P* < 0.001) (*Table 1*). This resulted in participants with CAD having a higher mean WMSI at rest (*P* < 0.001) and at peak stress (*P* < 0.001), and a higher burden of ischaemic segments (*P* < 0.001). ROC curve analysis for peak WMSI and ΔWMSI showed very high AUROC but not rest WMSI (*Figure 2A*).

### Artificial intelligence quantification of left ventricular volumes, left ventricular ejection fraction, and global longitudinal strain

Participants with significant CAD were more likely to have higher AI-calculated LV end-diastolic and end-systolic volumes, higher ischaemic dilatation ratios at rest and stress, and also were more likely to have an abnormal LVEF and GLS at both rest and peak SE (all *P* < 0.001 except for rest GLS *P* < 0.005, *Table 1*). ROC analysis (*Figure 2B* and *2C*) showed that peak LVEF and peak GLS were the best AI-calculated predictors for identifying participants with significant CAD with AUROCs of 0.86 and 0.82, respectively, with the optimal cut-offs for peak LVEF at 64% and peak GLS at −17.2%. The AUROCs for rest LVEF and rest GLS were much lower at 0.62 and 0.60, respectively, with the AUROCs for ΔLVEF and ΔGLS being intermediate between those at rest and at peak. Although the three AUROCs for peak, Δ and rest LVEF were similar to those of GLS, at the optimal cut-offs for all three, LVEF had a higher specificity and GLS had a higher sensitivity.

### Relationship between artificial intelligence-calculated left ventricular ejection fraction and global longitudinal strain and ischaemia

**Figure 3:**
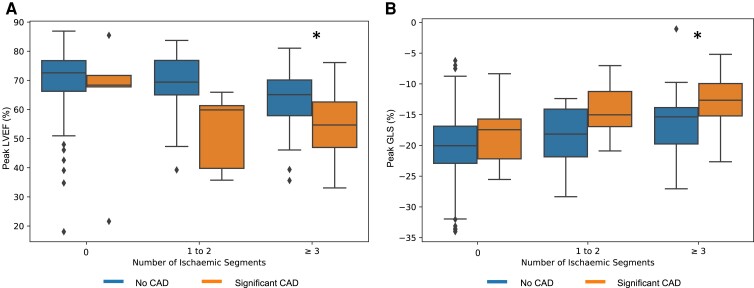
Box plots of artificial intelligence quantification of (*A*) left ventricular ejection fraction and (*B*) global longitudinal strain at peak stress stratified by ischaemic burden and presence of coronary artery disease. * *P* < 0.001 for ≥3 ischaemic segments; the number of cases for 0 and 1–2 ischaemic segments was too low for statistically assessing for significant differences.

**Figure 4:**
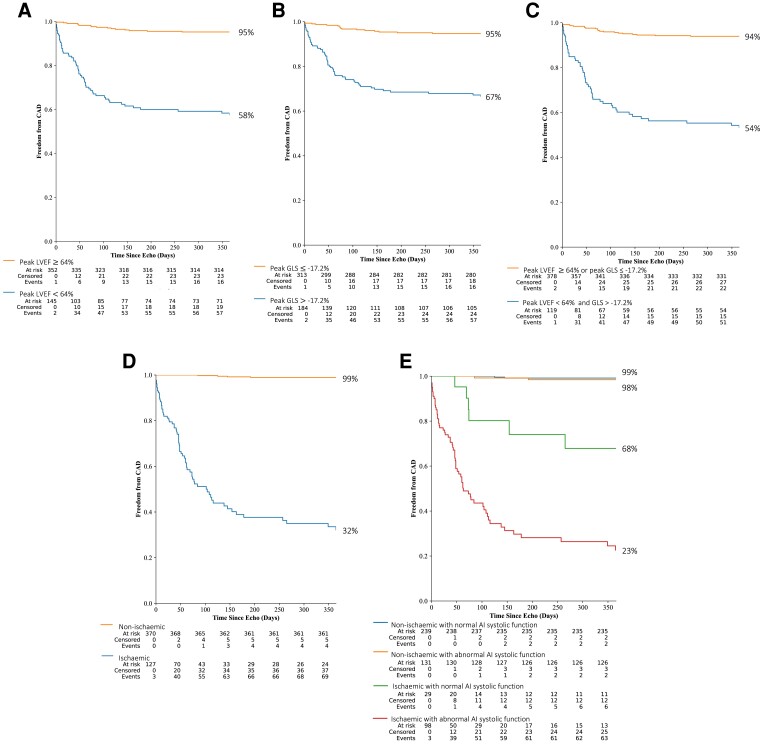
Kaplan–Meier cumulative event curves for freedom from significant coronary artery disease stratified according to artificial intelligence-calculated systolic function and/or the presence of inducible ischaemia over 12 months. (*A*) Artificial intelligence-calculated peak left ventricular ejection fraction. (*B*) Artificial intelligence-calculated peak global longitudinal strain. (*C*) Both artificial intelligence-calculated peak left ventricular ejection fraction and peak global longitudinal strain. (*D*) Ischaemia. (*E*) Ischaemia and both artificial intelligence-calculated peak left ventricular ejection fraction and peak global longitudinal strain; for brevity, normal artificial intelligence systolic function refers to peak left ventricular ejection fraction ≥64% or peak global longitudinal strain ≤17.2%, and abnormal artificial intelligence systolic function refers to both peak left ventricular ejection fraction <64% and peak global longitudinal strain >17.2%. Tables are shown below the graphs of participants at risk, censored and those who have had events for every 50 days, with the figures to the side of every event curve indicating the percentages after 12 months, with confidence intervals shown in Supplementary material online, *Table S4*.

The addition of AI-calculated LVEF and GLS demonstrated that patients with ≥3 ischaemic segments who were later found not to have significant CAD, had better LV systolic function at peak stress compared to patients with significant CAD (peak LVEF: 63 ± 10 vs. 55 ± 10%, *P* < 0.001; GLS: 16 ± 5 vs. 13.9 ± 4%, *P* < 0.001; *Figure 3*, respectively). Kaplan–Meier curves dichotomising the participants into groups with AI-calculated peak LVEF ≥64% or peak GLS ≤−17.2% showed that these groups had a lower proportion of patients free from significant CAD during the 12 months after SE (*Figure 3A*and*B*, Supplementary material online, *Table S4*). Although combining both of these measures of systolic function showed better separation of the Kaplan–Meier curves (*Figure 4C*), the separation was not as marked as having ischaemia (*Figure 4D*). However, combining abnormal AI-calculated systolic function with the presence of ischaemia was able to separate the ischaemic patients into groups of moderate and low proportions free from significant CAD (68 vs. 23%, *Figure 3E*).

### Combining clinical variables with artificial intelligence-calculated left ventricular ejection fraction and global longitudinal strain to identify significant coronary artery disease

In the univariable logistic regression analysis, age, male sex, body mass index, CAD risk factors, inducible ischaemia, and both AI-calculated peak LVEF and peak GLS significantly increased the odds of identifying significant CAD (*P* < 0.05 *Table 2*). A multivariable logistic regression model was then constructed using the clinical and SE outcomes (Model 1), and AI-calculated peak LVEF and peak GLS were then added into models individually as continuous variables (Models 2 and 3), in order to ascertain their incremental benefits. Due to high collinearity between the presence of inducible ischaemia and a moderate–severe ischaemic burden (≥3 segments), multivariable logistic regression was performed with the former. Following multivariable adjustment using Model 1, inducible ischaemia, hypercholesterolaemia and diabetes remained significant independent predictors of CAD (*Table 3*). AI-calculated peak LVEF and peak GLS were also significant independent predictors of CAD and significantly improved the *C*-statistic by 6–9% (*Table 3*, Supplementary material online, *Figure S2*).

**Table 2:** Clinical and stress echocardiography predictors of significant coronary artery disease from univariate regression

	Variable	OR	95% CI	*P*-value
**Clinical**	Age (years)	1.04	1.01–1.06	0.002
	Male sex	3.33	1.95–5.67	<0.001
	Body mass index	0.971	0.93–1.02	0.24
	Hypertension	1.64	1.00–2.71	0.05
	Hypercholesterolaemia	4.10	2.46–6.84	<0.001
	Diabetes mellitus	3.26	1.91–2.71	<0.001
**SE**	Ischaemia	106.75	37.58–303.20	<0.001
	≥3 ischaemic segments	43.76	22.38–85.55	<0.001
	Resting LVEF	0.95	0.93–0.97	<0.001
	Peak LVEF	0.88	0.86–0.91	<0.001
	Δ LVEF	1.10	1.07–1.12	<0.001
	Resting GLS	1.08	1.02–1.15	0.005
	Peak GLS	1.30	1.22–1.39	<0.001
	Δ GLS	1.16	1.10–1.22	<0.001

Odds ratios for quantitative predictors are shown for a unit increase. OR, odds ratio; CI, confidence interval; LVEF, left ventricular ejection fraction; GLS, global longitudinal strain.

**Table 3:** Multivariable predictors of significant coronary artery disease

		Model 1	Model 2	Model 3
**ORs for significant predictors**	Diabetes mellitus	2.94 (1.33–6.49)	3.98 (1.67–9.50)	3.44 (1.48–8.01)
	Hypercholesterolaemia	2.40 (1.20–4.78)	2.57 (1.23–5.37)	2.71 (1.32–5.59)
	Ischaemia	97.49 (37.10–218.91)	53.91 (21.80–133.30)	61.00 (24.80–149.70)
	Peak LVEF	—	0.93 (0.90–0.96)	—
	Peak GLS	—	—	1.15 (1.07–1.24)
**Model statistics**	*P*-value	<0.01	<0.01	<0.01
	*C*-statistic	0.78 (0.69–0.87)	0.83 (0.74–0.91)	0.84 (0.75–0.92)
	Sensitivity	61%	73%	76%
	Specificity	95%	94%	94%
	PPV	72%	72%	72%
	NPV	92%	94%	95%
	AIC	227	207	215

Odds ratios for quantitative predictors are shown for a unit increase. OR, odds ratio; PPV, positive predictive value; NPV, negative predictive value; AIC, Akaike information criterion; —indicated not applicable. C-statistic confidence intervals reflect the distribution of values from 2.5th to 97.5th percentiles from the bootstrapping analysis.

## Discussion

In this real-world SE study, we have shown that AI-calculated LVEF and GLS is able to provide independent and incremental predictive value to identify significant CAD beyond conventional risk factors and inducible ischaemia. A key strength of these findings is the increased availability of diagnostically relevant information without the requirement of advanced analysis skills or clinician time. Additionally, the incremental value of the automated analysis in this study included images acquired with ultrasound enhancing agents, potentially increasing the availability of quantitative assessment of LV systolic function during stress testing. The heterogeneity of sites, ultrasound systems, mode of stress, and contrast enhancement used in this study increases the likelihood that these findings are widely generalizable.

The implementation of AI tools within the clinical pathway has the potential to aid SE interpretation. Concerns about the reproducibility of semi-quantitative visual WMS in SE^9^ have given the impetus to investigate automated quantification of myocardial deformation in SE,^11^ principally using GLS because of its high inter-operator reproducibility.^9^ Automated and semi-automated speckle tracking provides an opportunity to obtain GLS and LVEF values and consider myocardial changes from underlying pathology; however, successful adoption in SE has been hampered by the poor reliability of speckles at higher heart rates and under contrast imaging. AI analysis pipelines delineate the endocardial boarder without the requirement for the tracking of image speckles or advanced clinician training for strain analysis. The benefits of such approaches include reductions in variability and workload but also the augmentation of diagnostic performance. In the present study, modelling the inclusion of AI-GLS and AI-LVEF within the clinical assessment of SE’s could increase diagnostic sensitivity by up to 15%. While the ability to contour and analyse contrast images is a distinct strength of the current study, further work is required to understand the performance and comparability of contrast-enhanced and non-contrast analyses separately. Using a larger sample of the current dataset, we have also demonstrated that the provision of an AI driven CAD classifier to interpreting clinicians can increase reader sensitivity by ∼10% on average.^6^ Thus, the coupling of automated analysis with AI CAD disease classifiers could serve as a second reader to positively augment diagnostic accuracy of SE.

A recent meta-analysis compared the utility of WMS to LV myocardial GLS assessed by tissue Doppler and manual contouring to derive speckle tracking. Use of WMS in the analysis had a lower pooled sensitivity than seen with WMS in the current study but similar specificity (0.83 vs. 0.86). The reported pooled sensitivity for GLS was also similar to the current study (0.88 vs. 0.82).^3^ The meta-analysis was based on several studies with small samples with multiple measurement methods, all of which were different to those used in this study, and there is an inherent risk of a publication bias in the meta-analysis. Additionally, the current study explicitly excluded those with a previous history of CAD who have a high pre-test probability.^1^ Given that a population without a previous history of CAD is better suited to the detection of inducible wall motion abnormalities, it is conceivable that WMS is more challenging and less reliable in those with a previous history of CAD.^12^ Therefore, this group may be more likely to benefit from quantification,^3^ particularly as GLS is largely determined by the contraction of longitudinal fibres that reside in the subendocardium,^13^ which itself is the myocardial layer most sensitive to ischaemia.^14^

Recent US and European guidelines recommend the use of quantitative echocardiography, specifically GLS, in the assessment of suspected or confirmed myocardial ischaemia in both the acute and elective settings and to direct clinical management.^15,16^ Non-invasive imaging for suspected CAD could be informative if it were able to reduce utilization of healthcare resources. This is potentially salient after findings favouring conservative patient management strategies (e.g. COURAGE^17^ and ORBITA,^18^) especially in cases of less severe myocardial ischaemia under stress.^10^ Although evidence from the FORECAST study^19^ did not find benefits for clinical outcome, it did report reductions in unnecessary invasive intervention, in agreement with CE-MARC2^20^. ^18^ In the current study, we demonstrate further risk stratification in those with inducible ischaemia on SE may be achievable through the use of quantitative measures of LVEF and GLS as they were able to separate patients with inducible ischaemia into two groups: one with an 11% increased risk of severe CAD and the other 36% lower risk.^21,22^ The results suggest that the AI assessment may help clinicians assess whether referral to invasive angiography in those with wall motion abnormalities could be reduced further based on severity of changes in quantitative measures, without the requirement of additional time or expertise. Our results also suggest that stress induced abnormal systolic function is potentially high (46%) even in the absence of regional wall motion changes. The long-term outcome for this patient group is of interest^23,24^ as they may benefit from further medical management and follow-up.

### Limitations

The study has a number of limitations. First, the method of CAD classification, whereby presence of inducible ischaemia was used to determine whether participants underwent coronary angiography, introduces a case selection bias for diagnosis of significant CAD. Furthermore, no central reading or quality control of readers was performed prior to entering data in the data bank, and our classification of CAD did not include data on fractional flow reserve. Second, additional case selection bias might also have been introduced by the retrospective nature of the RAINIER study. This results in high absolute AUROCs and odds ratio for diagnostic performance of WMSI but still allows relative evaluation of WMSI vs. LVEF and GLS. Third, our derivation of GLS utilizes only the apical two and four chamber views and the effect of including the three chamber view is unclear. Fourth, previous studies^1,3^ have shown that quantification of transient ischaemic dilatation is an independent predictor of mortality in patients with CAD^25–27^ and a marker of multivessel disease,^26^ whereas this study has focused on ischaemic dilatation at end-diastole and end-systole and shown they are useful for identifying significant CAD.^26^ Fifthly, those undergoing pre-operative assessment were excluded from this analysis and evaluation for this patient group would also be of interest. Finally, although we have demonstrated an incremental benefit of the use of LVEF and GLS in SE, we have not compared this increase in accuracy to our parallel developments in the use of AI to provide autonomous diagnostic assessment of the likelihood of CAD based on combinations of multiple parameters.^6^ We are currently conducting the multicentre PROspective randomised control Trial Evaluating the Use of AI in Stress echocardiography trial in 2500 participants (PROTEUS, ISRCTN registry ID 15113915) to evaluate the performance of the EchoGo platform^6^ for identifying significant CAD and on the rate of unnecessary angiography and healthcare costs. Furthermore, whether the use of GLS and LVEF may have value in those who do not achieve peak stress may be of interest to study.

## Conclusion

We have demonstrated that automated AI quantification of LVEF and GLS in contrast-enhanced and unenhanced SE images is feasible both at rest and with different modes of stress in a multicentre study. These measures conferred additional independent prognostic information in participants with suspected obstructive CAD, above and beyond inducible wall motion abnormalities alone. These findings support the increased use of quantification in SE in order to improve its diagnostic performance and utility in identifying and managing CAD. Future research is required to investigate the impact AI may have on predicting long-term adverse outcomes in patients undergoing echocardiography.

## Supplementary Material

oeac059_Supplemenatry_Data

## Data Availability

All data from the EVAREST prospective study (ClinicalTrials.gov Identifier: NCT03674255) and from Oregon Health Science University were used and collected under the terms of research agreements that do not allow the imaging and clinical data to be made available for public use. However, we have placed the AI derived data (LVEF and GLS) including patient-derived outcomes and the data analysis codes to a public repository on GitLab (https://gitlab.com/wpjhawkes/manuscript_codes/-/tree/master/Left_ventricular_assessment_with_artificial_intelligence_increases_the_diagnostic_accuracy_of_stress_echocardiography).

## Lead author biography

Dr Jamie O’Driscoll is a Reader in Cardiovascular Physiology at Canterbury Christ Church University and Clinical Scientist in Cardiology at St George’s University Hospitals NHS Foundation Trust. He has a keen research interest in the role of cardiac imaging, in particular the use of stress echocardiography in a range of clinical disease states including hypertension as well as valvular and cardiovascular disease. His recent work has focused on how cutting-edge artificial intelligence technology can be incorporated into the clinical practice of echocardiography.
