# Supplementary material for: Left ventricular assessment with artificial intelligence increases the diagnostic accuracy of stress echocardiography
Source: Eur Heart J Open. 2022 Sep 21;2(5):oeac059. doi: 10.1093/ehjopen/oeac059 (PMC9580364; doi:10.1093/ehjopen/oeac059)

Supplemental File

Left ventricular ejection fraction and global longitudinal strain calculated by artificial intelligence increases diagnostic accuracy of stress echocardiography

Jamie M. O'Driscoll[1,2,3*], William Hawkes[1*], Arian Beqiri[1], Angela Mumith[1], Andrew Parker[1], Ross Upton[1,4], Annabelle McCourt[4], William Woodward[4], Cameron Dockerill[4], Nikant Sabharwal[5], Attila Kardos[6], Daniel X Augustine[7,8], Katrin Balkhausen[9], Badrinathan Chandrasekaran[10], Soroosh Firoozan[11], Anna Marciniak[3], Stephen Heitner[12], Mrinal Yadava[12], Sanjiv Kaul[12], Rizwan Sarwar[1,4,13,14], Rajan Sharma[3], Gary Woodward[1] and Paul Leeson[4]

[1]Ultromics Ltd, 4630 Kingsgate, Cascade Way, Oxford Business Park South, Oxford, OX4 2SU.

[2]Canterbury Christ Church University, School of Psychology and Life Sciences, North Holmes Road, Kent, CT1 1QT.

[3]Department of Cardiology, St George's University Hospitals NHS Foundation Trust, Blackshaw Road, Tooting, London, SW17 0QT.

[4]Cardiovascular Clinical Research Facility, Radcliffe Department of Medicine, Division of Cardiovascular Medicine, University of Oxford, John Radcliffe Hospital, Oxford, UK.

[5]Oxford Heart Centre, Oxford University Hospitals NHS Foundation Trust, Oxford, UK.

[6]Department of Cardiology, Milton Keynes University Hospital NHS Foundation Trust, Milton Keynes, UK.

[7]Department of Cardiology, Royal United Hospitals NHS Foundation Trust, Bath, UK.

[8]Department for Health, University of Bath, Bath, UK.

[9]Department of Cardiology, Royal Berkshire NHS Foundation Trust, Reading, UK.

[10]Department of Cardiology, Great Western Hospitals NHS Foundation Trust, Swindon, UK.

[11]Department of Cardiology, Buckinghamshire Healthcare NHS Trust, High Wycombe, UK.

[12]Knight Cardiovascular Institute, Oregon Health & Science University, Portland, Oregon, USA.

[13]Experimental Therapeutics, Radcliffe Department of Medicine, University of Oxford, John Radcliffe Hospital, Oxford, UK.

[14]Cardiology Department, Oxford University Hospitals NHS Foundation Trust, Oxford, UK.

Running title: AI-calculated LVEF and GLS in stress echocardiography

Correspondence to: Professor Paul Leeson, Oxford Cardiovascular Clinical Research Facility, RDM Division of Cardiovascular Medicine, John Radcliffe Hospital, Oxford. OX3 9DU. E-mail: paul.leeson@cardiov.ox.ac.uk

Contents

Supplementary Figure 1. Study flow diagram.

Supplementary Table 1. Wall motion score reproducibility.

Supplementary Table 2. Extent, significance and management of significant CAD in study population.

Supplementary Table 3. Haemodynamic response during SE.

Supplementary Table 4. Freedom from significant CAD between different participant groups over the 12 months.

Supplementary Figure 2. Comparing of the incremental benefit of incorporating AI-calculated LV systolic function at peak stress to standard clinical assessment using bootstrapping of logistic regression models.

Supplementary Figure 1. Study flow diagram.

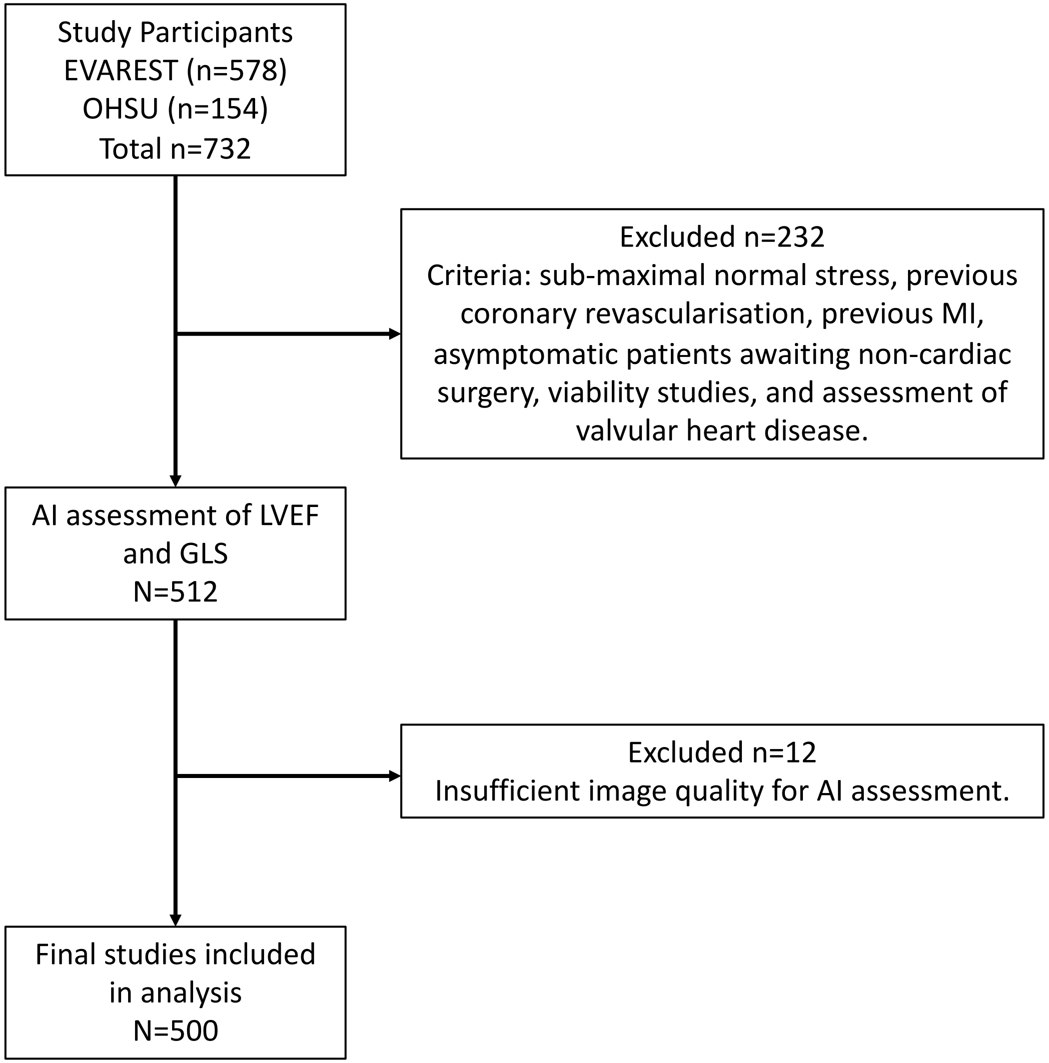

Supplementary Table 1. Wall motion score reproducibility.

	All<br>(n = 20)<br>ICC (95% CI)	Ischaemic only<br>(n=12)<br>ICC (95% CI)
**Inter-operator variability**	0.88 (72, 95)	0.76 (35,92)
**Intra-operator variability**	0.94 (0.86, 0.98)	0.88 (0.64, 0.96)

Supplementary Table 2. Extent, significance and management of significant CAD in study population. PCI, percutaneous coronary intervention. CABG, coronary artery bypass graft.

		All	Non-ischaemic (*n*=370)		Ischaemic (*n*=130)	
			Normal AI systolic function	Abnormal AI systolic function	Normal AI systolic function	Abnormal AI systolic function
***n*, (%age of participants)**		500 (100%)	239 (48%)	131 (26%)	29 (48%)	101 (20%)
**Angiography, *n* (%age)**		118 (24%)	5 (3%)	6 (5%)	18 (62%)	89 (88%)
**Significant CAD, *n* (%age of angiograms)**		74 (63%)	2 (40%)	2 (33%)	6 (33%)	64 (63%)
**Vessels with significant CAD, *n* (%age of significant CAD)**	1	34 (29%)	0 (0%)	1 (50%)	4 (67%)	29 (45%)
	2	30 (25%)	2 (100%)	0 (0%)	1 (17%)	27 (42%)
	3	10 (8%)	0 (0%)	1 (50%)	1 (17%)	8 (13%)

Supplementary Table 3. Haemodynamic response during SE. HR, heart rate; APMHR, age-predicted maximum HR, sBP, systolic blood pressure; dBP, diastolic blood pressure; RPP, rate-pressure product

			**No CAD (*n*=426)**	**Significant CAD (*n*=74)**	***P*-value**
**All SEs**	HR (beats/min)	Baseline	76 ± 14	74 ± 13	0.238
		Peak	140 ±14	132 ± 17	**<0.001**
		%age APMHR	89 ± 8	87 ± 10	0.079
	sBP (mmHg)	Baseline	140 ± 21	142 ± 21	0.434
		Peak	150 ± 33	169 ± 30	**<0.001**
	dBP (mmHg)	Baseline	77 ± 13	78 ± 12	0.759
		Peak	74 ± 16	80 ± 16	**0.004**
	Peak RPP (beats·mmHg/min)		21101 ± 5258	22482 ± 5037	**0.04**
**Dobutamine SEs**	HR (beats/min)	Baseline	77 ± 14	73 ± 14	0.2
		Peak	138 ± 14	132 ± 12	**0.009**
		%age APMHR	89 ± 9	88 ± 9	0.574
	sBP (mmHg)	Baseline	141 ± 22	141 ± 27	0.065
		Peak	141 ± 27	158 ± 29	**0.002**
	dBP (mmHg)	Baseline	76 ± 13	78 ± 14	0.518
		Peak	70 ± 15	73 ± 11	0.384

	Peak RPP (beats.mmHg/min)		19458 ± 4033	20791 ± 4495	0.09
**Exercise SEs**	HR (beats/min)	Baseline	74 ± 14	75 ± 14	0.975
		Peak	146 ± 15	134 ± 21	**<0.001**
		%age APMHR	91 ± 8	87 ± 12	**0.024**
	sBP (mmHg)	Baseline	136 ± 16	137 ± 18	0.640
		Peak	180 ± 34	177 ± 28	0.716
	dBP (mmHg)	Baseline	80 ± 11	77 ± 10	0.236
		Peak	87 ± 14	86 ± 16	0.694
	Peak RPP (beats.mmHg/min)		26150 ± 5146	23679 ± 5108	**0.024**

Supplementary Table 4. Freedom from significant CAD between different participant groups over the 12 months. Participant groups are shown as graphed in the Kaplan-Meier curves shown in Figure 3. Normal AI systolic function at peak is defined as peak GLS ≤ or peak LVEF ≥ 64%; abnormal AI systolic function at peak is defined as peak GLS > -17.2% and peak LVEF < 64%. CI, confidence interval

Figure	Participant group	%age	95% CI	*P*-value
3A	Peak LVEF ≥ 64%	95%	92% – 97%	<0.001
	Peak LVEF < 64%	58%	49% – 65%	
3B	Peak GLS ≤ 17.2%	95%	92% – 98%	0.007
	Peak GLS > 17.2%	67%	59% – 73%	
3C	Normal AI systolic function at peak	94%	91% – 96%	<0.001
	Abnormal AI systolic function at peak	54%	43% – 62%	
3D	Non-ischaemic	99%	97% – 100%	0.01
	Ischaemic	32%	23% – 42%	
3E	Non-ischaemic with normal AI systolic function at peak	99%	97% – 100%	<0.001
	Non-ischaemic with abnormal AI systolic function at peak	98%	94% – 100%	
	Ischaemic with normal AI systolic function at peak	68%	42% – 84%	
	Ischaemic with abnormal AI systolic function at peak	23%	14% – 33%	

Supplementary Figure 2. Comparing of the incremental benefit of incorporating AI-calculated LV systolic function at peak stress to standard clinical assessment using bootstrapping of logistic regression models.

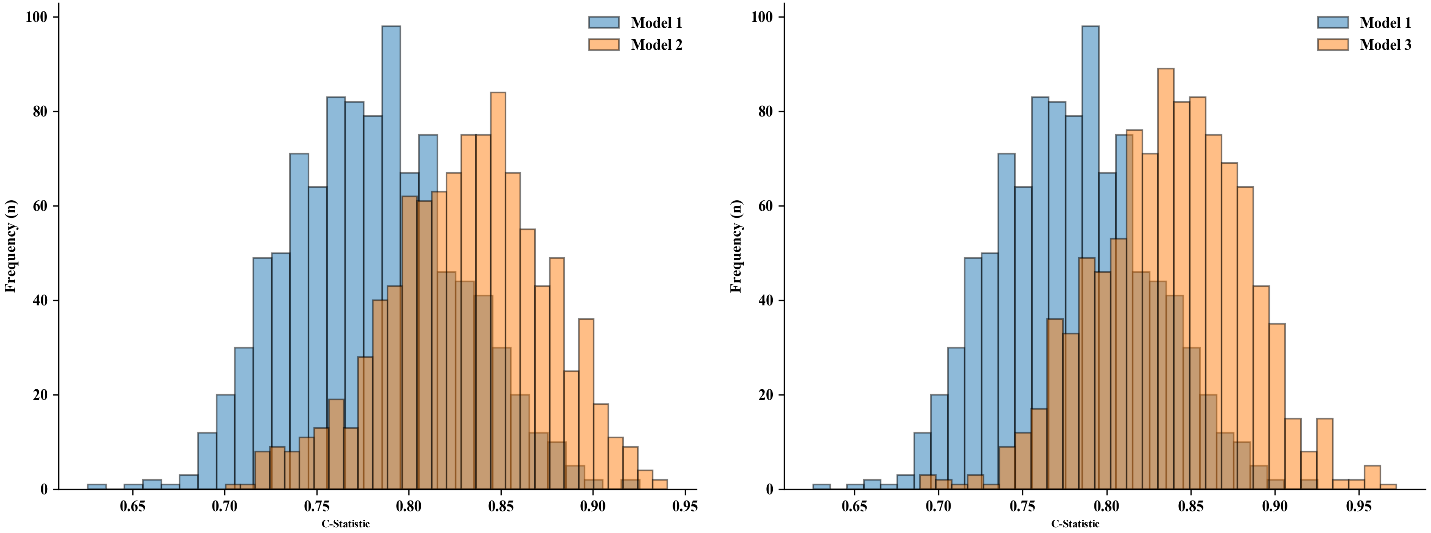

Supplement: oeac059_Supplemenatry_Data [file oeac059_supplemenatry_data.pdf]
